# Geiparvarin Inhibits OS Metastasis through Upregulation of ANGPTL4 Expression by Inhibiting miRNA-3912-3p Expression

**DOI:** 10.1155/2022/4663684

**Published:** 2022-04-12

**Authors:** Fuling Jiang, Guang-Jie Wang, Ping Huang, Shu Chen, He Xiao, Liang Zhang, Hua Zou

**Affiliations:** ^1^Department of Spine Surgery, Center of Orthopedics, Daping Hospital, Army Medical University, Chongqing 400042, China; ^2^Department of Oncology, The General Hospital of Western Theater Command, Chengdu, Sichuan 610083, China; ^3^Department of Radiation Oncology, Chongqing University Cancer Hospital, Chongqing 400033, China; ^4^Cancer Center, Daping Hospital, Army Medical University, Chongqing 400042, China

## Abstract

**Background:**

Geiparvarin (GN) is a natural compound with anticancer activity. However, the effect of GN on osteosarcoma (OS) and the anticancer mechanism of GN are still unclear.

**Methods:**

Cell viability was measured by MTT assay. Invasion and migration were measured by transwell assay. The miRNAs, genes, and signaling pathways affected by GN were confirmed by whole-genome sequencing and bioinformatics analysis. The expression level of mRNA and protein was measured by qRT-PCR and western blot. Animal experiment was performed for confirming the GN anticancer effect and side effect *in vivo*.

**Results:**

Our results show that GN significantly inhibits OS cell growth and metastasis *in vitro*. *In vivo* experiment also showed that GN dramatically suppressed OS lung metastasis and no side effects were found. GN treatment inhibited OS metastasis through upregulating the ANGPTL4 expression. In addition, GN inhibited the expression of miR-3912-3p, which targets ANGPTL4.

**Conclusion:**

Our data clearly indicate that GN is a candidate drug for OS treatment, and GN plays its role through miR-3912-3p/ANGPTL4 in OS.

## 1. Introduction

Osteosarcoma (OS) is a rare malignancy of bone, but it is the most common primary malignant tumor in children and adolescents [1]. OS patients are typically treated with surgery and intensive adjuvant chemotherapy, and this treatment method was adopted in the 1970s and has been used until now [2]. However, treatment of OS often fails due to chemoresistance development [3]. At present, the OS chemotherapy mainly relies on the methotrexate, cisplatin, doxorubicin, and ifosfamide [4]. Thus, once patients develop resistance to these drugs, there is no alternative drug, which means that the patients' prognosis is poor, indicating further research is needed to development new agents for OS treatment.

MicroRNAs (miRNAs) are small noncoding RNAs that inhibit gene expression by cleavaging mRNA or suppressing translation through interaction with complementary sequences in the 3-UTRs of the target gene mRNA [5]. Dysregulation of miRNAs has been demonstrated in most cancers including OS [6], and these dysregulated miRNAs play a crucial role during OS metastasis [7] and chemoresistance development [8]. Notably, targeting these dysregulated miRNAs can overcome chemoresistance and inhibit OS lung metastasis [9], indicating these miRNAs are also an important therapeutic target and interfering the levels of these abnormally expressed miRNAs is a strategy for the OS treatment.

In the past few decades, agents derived from natural sources have received extensive attention in cancer treatment research due to their safety and efficacy [10]. However, these natural agents have not been popularly accepted because their molecular mechanisms are poorly defined [11]. Studies show that natural agents exhibit their anticancer effects through many different mechanisms, including affecting miRNAs expression in cancer [12]. Thus, regulating miRNA by natural agents becomes a new strategy for cancer treatment [13]. GN is a natural compound isolated from the leaves of *Geijera parviflora* and exhibits anticancer activity in various types of cancer [14]. However, the inhibitory effect of GN on OS and its effect on miRNA expression has not been reported yet. Thus, in this study, we investigated the GN anti-OS effects and whether GN plays its function by affecting the levels of miRNAs.

Our results show that GN exhibits anticancer activity in OS. GN affected many miRNA expression and altered various signaling pathways related to cancer. Among them, upregulation of ANGPTL4 by downregulating miR-3912-3p is an anti-OS mechanism of GN.

## 2. Materials and Methods

### 2.1. Materials

OS cell line HOS, lung cancer cell lines A549 and H522, and gastric cancer cell lines AGS and SGC-7901 were obtained from the American Type Culture Collection (Manassas, VA, USA). OS cell line 143B was purchased from the Chinese Academy Sciences Cell Bank of Type Culture Collection (Shanghai, China). All cell culture-related materials were obtained from Sigma (St. Louis, MO, USA). GN (GN) and transwell chambers were purchased from Unigen, Inc. (Seattle, WA, USA) and Costar (Cambridge, MA, USA), respectively. The MTT kit and luciferase activity detection kit were obtained from Beyotime Biotechnology (Shanghai, China) and Promega (Madison, WI, USA), respectively. All antibodies that were used in this study were purchased from Abcam (Cambridge, MA, USA). Enhanced chemiluminescence detection kit, TRIzol RNA extraction kit, qRT-PCR kit, and lipofectamine 3000 were obtained from Thermo Fisher Scientific (Carlsbad, CA, USA).

### 2.2. Cell Culture and Cell Viability Assay

All cells were cultured in a Dulbecco's modified Eagle's medium with 10% fetal bovine serum at 37°C in a humidified atmosphere containing 5% CO_2_. For cell viability assay, indicated cells were seeded in 96-well plate at density of 5000 cells per well. After 12 hours of cell seeding, cells were treated with indicated drugs for indicated times and then cell viability assay was performed using the MTT assay kit according to manufacturer's instruction.

### 2.3. Transwell Assay

Cells were treated with GN for 48 h, and then 10000 cells in a serum-free medium were reseeded in the upper wells of chambers. The lower chambers contained the medium with 10% fetal bovine serum. After 24 h of reseeding, cells in the upper wells of chambers were removed and the invaded cells were fixed with 2.5% glutaraldehyde and stained with crystal violet, photographed, and counted.

### 2.4. Western Blot Analysis

30 µg of proteins from cells was separated by sodium dodecyl sulfate-polyacrylamide gel electrophoresis and transferred to nitrocellulose membranes. The membranes were washed with Tris-buffered saline with Tween 20 (TBST) and blocked with 5% skim milk. Then, membranes were incubated with primary antibodies in 5% skim milk overnight at 4°C. The membranes were washed with TBST and incubated with secondary antibody for 1 h at room temperature. The protein bands were visualized with an enhanced chemiluminescence detection kit.

### 2.5. Luciferase Assay

Firefly luciferase reporter constructs containing the mutant or wild-type 3-UTR of ANGPTL4 and Renilla luciferase plasmid were cotransfected with indicated cells with lipofectamine 3000. After 24 h of transfection, cells were transfected with indicated miRNA or negative control oligonucleotides. After 72 h of miRNA transfection, cells were lysed and subjected to luciferase activity measurement. The luciferase activity was measured by a dual-luciferase reporter assay system according to manufacturer's instruction.

### 2.6. Whole Genome Sequencing and qRT-PCR

Indicated cells were treated with GN or PBS for indicated time periods, and then RNAs were isolated using a TRIzol RNA extraction kit. The whole-genome sequencing and bioinformatics analysis were performed by Gene Denovo Biotechnology Co. (Guangzhou, China). The mRNA expression of ANGPTL4 and GAPDH was detected by the qRT-PCR kit according to manufacturer's instruction, and the relative expression of ANGPTL4 was normalized to the GAPDH expression. The primers used for ANGPTL4 amplification were 5′-GGCTCATGTTACTTCAACCG-3′ and 5′-CCGTGATGCTATGCACCTTCT-3′; the primers used for GAPDH amplification were 5′-GGAGCGAGATCCCTCCAAAAT-3′ and 5′-GGCTGTTGTCATACTTCTCATGG-3′.

### 2.7. Animal Experiments

OS lung metastasis models were generated using 143B cells. 1 × 10^6^ cells in 100 *μ*l PBS were injected intravenously into the tail vein of the 6-week-old female nude mice (*n* = 6 per group). After one week of the cell injection, the mice were randomly divided into two groups. The control group mice were treated with PBS, and another group were treated with GN (5 mg/kg body weight) by IP injection once every 3 days for 4 weeks. The body weight of the mouse was measured every 1 week. This animal experiment complied with the Daping Hospital, Army Medical University Policy on the Care and Use of Laboratory Animals.

### 2.8. Statistical Analysis

Less than 0.05 was considered statistically significant, and all values are presented as the mean ± standard deviation. Student's *t*-test was used to evaluate the comparisons between control and treatment groups. All statistical analyses were performed using Prism software (GraphPad).

## 3. Results

### 3.1. GN Significantly Inhibits Cancer Cells Viability

**Figure 1:**
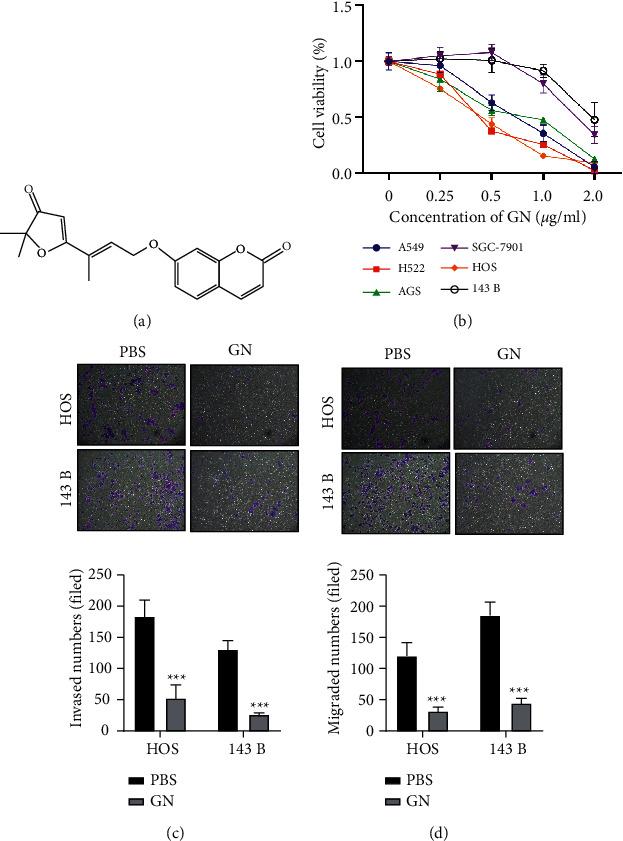
GN significantly inhibits cancer cells. (a) Pharmaceutical chemical structure of GN. (b) Indicated cells were treated with various concentrations of GN for 48 h and the cell viability was measured by MTT assay. (c) OS cells were treated with GN or PBS for 48 h and performed invasion assay. (d) OS cells were treated with GN or PBS for 48 h and performed migration assay. 143B and HOS cells were treated with 1 *μ*g/ml and 1.2 *μ*g/ml GN, respectively. *∗∗∗p* < 0.001.

First, we confirmed the potential anticancer effect of GN (Figure 1(a)) in several types of cancer cell lines by cell viability assay, including non-small-cell lung cancer, gastric cancer, and OS. Our results showed that GN significantly inhibited all types of cancer cells in a concentration-dependent manner, and the IC50 value was between 0.7 and 1.8 µg/mL (Figure 1(b)). In addition, GN significantly inhibited invasion (Figure 1(c)) and migration of OS cells when cells were treated with IC50 concentration for 48 hours (Figure 1(d)). These findings indicate that GN can inhibit OS metastasis.

### 3.2. Identification of Differentially Expressed Genes and miRNAs by GN in OS

**Figure 2:**
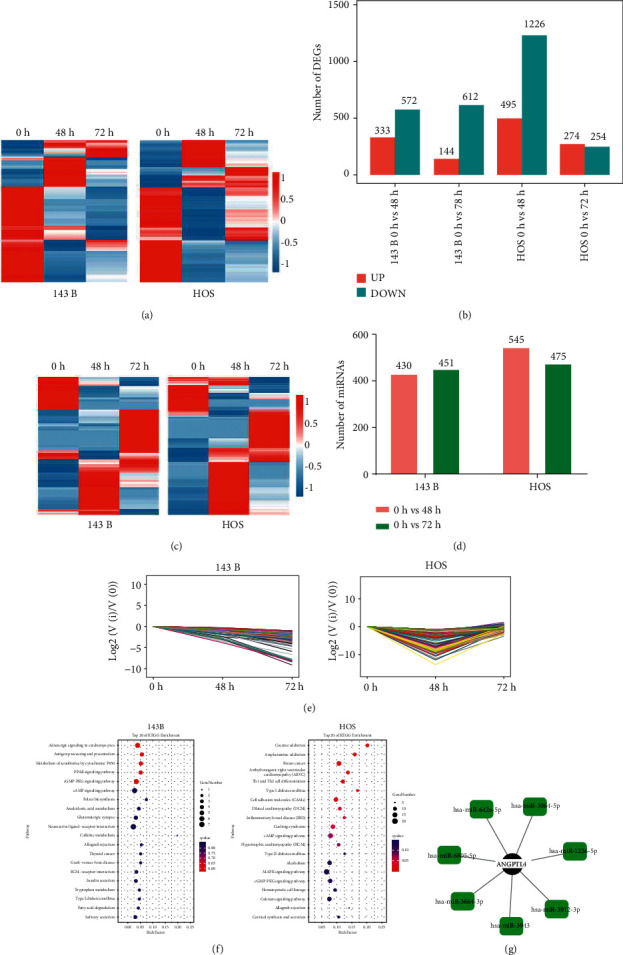
GN treatment altered many miRNAs and gene expressions in OS cells. (a) Heatmap showing genes whose mRNA expression level was altered by GN treatment. (b) Bar graph showing that up- or downregulated gene numbers by GN in different OS cells at different treatment time periods. (c) Heatmap showing miRNAs whose expression level was altered by GN treatment. (d) Bar graph showing the miRNA numbers whose expression was affected by GN in different OS cells. (e) Significant clustered genes profile that was affected by GN in OS cells. (f) KEGG gene set enrichment analysis showing enriched pathways of miRNA regulated differentially expressed genes (DEGs) that were identified in OS cells. (g) Network of mRNA-microRNA visualized by Cytoscape. The mRNA expression of genes in the red rectangular may be regulated by microRNAs surrounding them.

To investigate the anticancer mechanism of GN, we performed whole genome sequencing using OS cells that were treated with GN or vehicles. A large number of genes were identified as differentially expressed genes (DEGs) in OS cell lines treated with GN after 48 hours and 72 hours in comparison with control (Figure 2(a)). Specifically, 333 and 572 genes were upregulated and downregulated in 143B cell line treated with GN after 48h, whereas 144 and 612 genes were upregulated and downregulated after 72 h (Figure 2(b)). The 495 and 1226 genes upregulated and downregulated by GN were observed in HOS cells treated with GN after 48 hours and 274 and 254 genes in 72 hours (Figure 2(b)). Meanwhile, we detected 430 and 451 species of miRNAs that differentially expressed 48 hours and 72 hours after GN treatment in 143B cells, whereas 545 and 475 species of miRNAs that differentially expressed 48 hours and 72 hours after GN treatment were identified, respectively, in HOS cell line (Figures 2(c) and 2(d)). Together, these findings suggest that GN may play its role through the miRNA-mRNA network in OS.

### 3.3. Trend Analysis and miRNA-mRNA Interaction Network

In order to elucidate the miRNA-mRNA interaction network induced by the treatment of GN, DEGs were first associated with miRNA species identified through differential analysis with fold-change direction opposite to those of DEGs. From these candidate genes, trend analysis was used to further narrow DEGs. As shown in Figure 2(e), genes in 143B cell line and in HOS cell line were significantly clustered. KEGG analysis showed that these genes that may be regulated by miRNAs in 143B and HOS cell lines are involved in the regulation of multiple signaling pathway (Figure 2(f)). However, taking overlapped pathways into consideration, the ANGPTL4 miRNA-mRNA network was the only enriched pathway regardless of duration of treatment and cell lines used (Figure 2(g)), indicating that ANGPTL4 plays a crucial role in the anti-OS effect of GN.

### 3.4. GN Plays Its Anticancer Role through Upregulation of ANGPTL4 by Inhibiting miR-3912-3p in OS Cells

**Figure 3:**
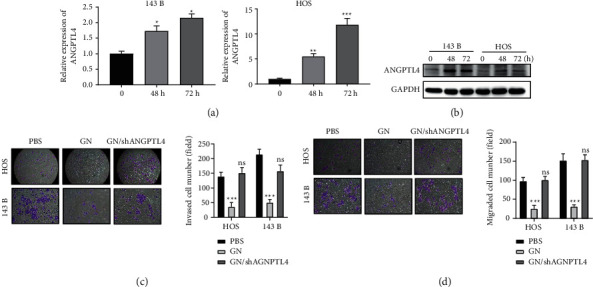
GN plays its anticancer role through upregulating ANGPTL4 in OS. (a) mRNA expression level of ANGPTL4 was measured by qRT-PCR in OS after GN treatment. (b) Protein expression level of ANGPTL4 was measured by Western blot analysis in OS after GN treatment. (c) Silencing of ANGPTL4 blocked anti-invasion effect of GN in OS cells. (d) Silencing of ANGPTL4 blocked anti-invasion effect of GN in OS cells. *∗∗∗p* < 0.001; ns, not significant.

**Figure 4:**
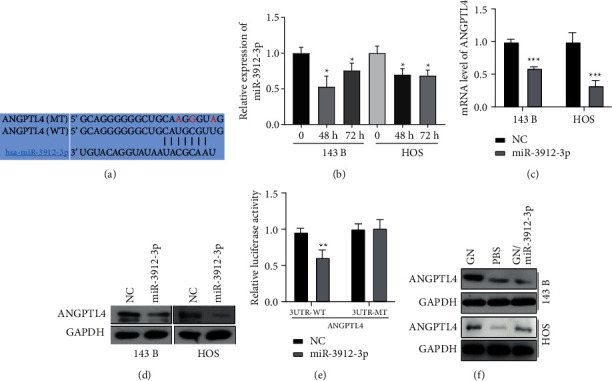
ANGPTL4 is a target of miR-3912-3p in OS. (a) Predicted binding sites of miR-3912-3p in the wild-type 3-UTR of ANGPTL4. Mutations in this 3-UTR are highlighted in red. (b) Inhibitory effects of GN on miR-3912-3p expression were measured by qRT-PCR analysis. (c) Inhibitory effects of miR-3912-3p on ANGPTL4 mRNA expression was measured by qRT-PCR analysis. (d) Inhibitory effects of miR-3912-3p on ANGPTL4 protein level was measured by western blot. (e) Luciferase activity of the reporter driven by the wild-type or mutant 3-UTRs of ANGPTL4 in OS cells cotransfected with control oligonucleotides (NC) or miR-3912-3p mimics. (f) miR-1392-3p effects on GN induced upregulation of ANGPTL4 was measured by western blot.

To investigate whether ANGPTL4 directly increased by GN treatment, the OS cells were treated with GN and detected ANGPTL4 expression by qRT-PCR and western blot. Our results show that GN treatment significantly increased ANGPTL4 expression in HOS and 143B cell lines at both mRNA (Figure 3(a)) and protein levels after 48 h and 72 h treatment (Figure 3(b)). In addition, GN-inhibited invasion and migration of OS cells were blocked by silencing of ANGPTL4 (Figure 3(c) and 3(d)), suggesting that GN plays its anticancer role in OS through upregulation of ANGPTL4. Then, we investigate which miRNAs were involved in regulation of ANGPTL4 expression when OS cells were treated with GN. Among the candidate miRNAs (Figure 2(g)), miRNA database (targetscan.org) identified that miR-3912-3p may target 3′-UTR of ANGPTL4 (Figure 4(a)). Thus, we investigated whether miR-3912-3p was inhibited by GN treatment in OS cells. As shown in Figure 4(b), GN treatment inhibited miR-3912-3p levels in both 143B and HOS cell lines. In addition, our mRNA and western blot analyses show that miR-3912-3p negatively regulates ANGPTL4 expression in OS cells at both mRNA and protein levels (Figures 4(c) and 4(d)). Furthermore, we identified that miR-3912-3p overexpression inhibits luciferase expression that is regulated by 3-UTR of ANGPTL4, while the expression of luciferase regulated by mutant 3-UTR of ANGPTL4 in the miR-3912-3p binding site is not affected by miR-3912-3p (Figure 4(e)), suggesting that miR-3912-3p inhibits ANGPTL4 by directly binding to 3-UTR. Notably, GN-upregulated expression of ANGPTL4 was blocked by miR-3912-3p overexpression (Figure 4(f)), suggesting that GN upregulates ANGPTL4 through inhibiting miR-3912-3p expression in OS.

### 3.5. GN Significantly Inhibits OS Lung Metastasis In Vivo

**Figure 5:**
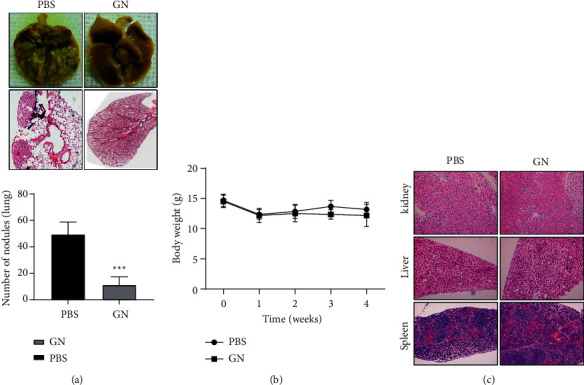
GN significantly inhibited OS lung metastasis. (a) GN treatment inhibited OS lung metastasis in vivo. (b) GN treatment did not affect body weight of mice. (c) Representative H&E staining of various organs.

Finally, using animal models we confirmed the anti-metastatic effects of GN in OS. Lung metastasis models were generated by tail vein injection of 143B cells into nude mice; then, GN was injected through intraperitoneal injection once every 3 days. Our results show that GN can significantly inhibit OS lung metastasis. As shown in Figure 5(a), animals in the GN treatment group had fewer tumor nodules and smaller tumor sizes compared to the vehicle treatment group. Notably, GN treatment did not affect the body weight of animals (Figure 5(b)) and the pathological examination also showed that the GN did not cause any lesions on the major organs of animals (Figure 5(c)), suggesting that GN is a candidate drug for the treatment of OS without side effects.

## 4. Discussion

The anticancer effects of GN and its analogs were demonstrated in various types of cancer [15]. However, the effects of GN in OS have not been reported. Here, a series of *in vitro* and *in vivo* experiments clearly indicated that GN also has a strong inhibitory effect on OS, especially for inhibition of OS lung metastasis. Furthermore, in animal experiments, we did not find that GN caused side effects on experimental animals. Taken together, these findings suggest that GN has the potential to become a drug for OS treatment.

Although GN has a significant biological activity on various human tumors, the underlying mechanism is still unclear [15]. Here, we revealed the anti-OS mechanism of GN. Natural compounds from plants play a role through many different mechanisms [16]. Among them, regulating the expression of miRNAs is also an important anticancer mechanism of natural compounds [12]. In the present study, we report for the first time that GN altered expression of many miRNAs, thereby affecting various signaling pathways that are related to cancer. Among them, miR-3912-3p is one of the significantly downregulated miRNAs by GN in OS and the overexpression of miR-3912-3p significantly blocked GN-induced anti-metastasis effect in OS. In addition, we demonstrated that ANGPTL4 is a target gene of miR-3912-3p and upregulated by GN in OS cells. Notably, silencing of ANGPTL4 blocked GN-induced anti-OS metastasis effects. Together, these findings suggest that GN inhibits OS through upregulating ANGPTL4 expression by downregulating miRNA-3912-3p. However, the mechanism by which GN regulates the expression of miRNAs in OS is still unclear and needs further study.

ANGPTL4 is a member of the angiopoietin-like protein family. Recent studies show that ANGPTL4 expression was dysregulated in various cancers and affects cancer progression. However, the role of ANGPTL4 in cancers is controversial because studies reported contrary effects of ANGPTL4 in cancers [17], even in the same cancer [17, 18]. Cai et al. report that contrary effects of ANGPTL4 in cancer were caused by different subtypes of ANGPTL4 [17]. Native full-length ANGPTL4 produces the COOH-terminal fibrinogen-like fragment and the N-terminal coiled-coil domain via proteolytic processing [17, 19]. In breast cancer, full-length ANGPTL4 was associated with lower relapse and vascular invasion rates and overexpression of full-length ANGPTL4 inhibited breast cancer cell adhesion and attachment, which lead to inhibition of cell invasion and migration [17]. In present study, we also demonstrated the anti-invasion and anti-metastasis effects of full-length ANGPTL4 in OS.

## 5. Conclusions

In summary, GN is a candidate drug for OS treatment and plays its role by altering the miRNA expression level. Among them, downregulating miR-3912-3p lead to upregulation of ANGPTL4, thereby showing that inhibition of OS metastasis is one anticancer mechanism of GN.

## Data Availability

All original information of this study is provided within the article. For further details, please contact the corresponding author.

## References

[1] Messerschmitt P. J., Garcia R. M., Abdul-Karim F. W., Greenfield E. M., Getty P. J. (2009). Osteosarcoma. *Journal of the American Academy of Orthopaedic Surgeons*.

[2] Kansara M., Teng M. W., Smyth M. J., Thomas D. M. (2014). Translational biology of osteosarcoma. *Nature Reviews Cancer*.

[3] Huang J., Ni J., Liu K. (2012). HMGB1 promotes drug resistance in osteosarcoma. *Cancer Research*.

[4] Meyers P. A., Schwartz C. L., Krailo M. (2005). Osteosarcoma: a randomized, prospective trial of the addition of ifosfamide and/or muramyl tripeptide to cisplatin, doxorubicin, and high-dose methotrexate. *Journal of Clinical Oncology*.

[5] Bartel D. P. (2004). MicroRNAs. *Cell*.

[6] Zhang J., Yan Y.-G., Wang C., Zhang S.-J., Yu X.-H., Wang W.-J. (2015). MicroRNAs in osteosarcoma. *Clinica Chimica Acta*.

[7] Xu M., Jin H., Xu C.-X. (2015). miR-382 inhibits osteosarcoma metastasis and relapse by targeting Y box-binding protein 1. *Molecular Therapy*.

[8] Xu M., Jin H., Xu C.-X. (2014). miR-382 inhibits tumor growth and enhance chemosensitivity in osteosarcoma. *Oncotarget*.

[9] Wang S.-N., Luo S., Liu C. (2017). miR-491 inhibits osteosarcoma lung metastasis and chemoresistance by targeting *α*B-crystallin. *Molecular Therapy*.

[10] Gupta S. C., Prasad S., Sethumadhavan D. R. (2013). Nimbolide, a limonoid triterpene, inhibits growth of human colorectal cancer xenografts by suppressing the proinflammatory microenvironment. *Clinical Cancer Research*.

[11] Zhao Z., Jia Q., Wu M.-S. (2018). Degalactotigonin, a natural compound from Solanum nigrum L, inhibits growth and metastasis of osteosarcoma through GSK3*β* inactivation-mediated repression of the hedgehog/gli1 pathway. *Clinical Cancer Research*.

[12] Ahmed F., Ijaz B., Ahmad Z., Farooq N., Sarwar M. B., Husnain T. (2020). Modification of miRNA Expression through plant extracts and compounds against breast cancer: mechanism and translational significance. *Phytomedicine: International Journal of Phytotherapy and Phytopharmacology*.

[13] Sethi S., Li Y., Sarkar F. (2013). Regulating miRNA by natural agents as a new strategy for cancer treatment. *Current Drug Targets*.

[14] Bocca C., Gabriel L., Miglietta A. (2001). Cytoskeleton-interacting activity of geiparvarin, diethylstilbestrol and conjugates. *Chemico-Biological Interactions*.

[15] Zhang Y., Lv Z., Zhong H. (2012). Convenient synthesis of novel geiparvarin analogs with potential anti-cancer activity via click chemistry. *European Journal of Medicinal Chemistry*.

[16] Talib W. H., Alsalahat I., Daoud S., Abutayeh R. F., Mahmod A. I. (2020). Plant-derived natural products in cancer research: extraction, mechanism of action, and drug formulation. *Molecules*.

[17] Cai Y. C., Yang H., Wang K. F., Chen T. H., Jiang W. Q., Shi Y. X. (2020). ANGPTL4 overexpression inhibits tumor cell adhesion and migration and predicts favorable prognosis of triple-negative breast cancer. *BMC Cancer*.

[18] Zhao J., Liu J., Wu N. (2020). ANGPTL4 overexpression is associated with progression and poor prognosis in breast cancer. *Oncology Letters*.

[19] Lei X., Shi F., Basu D. (2011). Proteolytic processing of angiopoietin-like protein 4 by proprotein convertases modulates its inhibitory effects on lipoprotein lipase activity. *Journal of Biological Chemistry*.

